# Amino acid substitution equivalent to human chorea-acanthocytosis I2771R in yeast Vps13 protein affects its binding to phosphatidylinositol 3-phosphate

**DOI:** 10.1093/hmg/ddx054

**Published:** 2017-03-01

**Authors:** Weronika Rzepnikowska, Krzysztof Flis, Joanna Kaminska, Marcin Grynberg, Agnieszka Urbanek, Kathryn R. Ayscough, Teresa Zoladek

**Affiliations:** 1 Institute of Biochemistry and Biophysics, Polish Academy of Sciences, Pawińskiego 5a, 02-106 Warsaw, Poland; 2 Department of Biomedical Science, University of Sheffield, Sheffield S10 2TN, UK

## Abstract

The rare human disorder chorea-acanthocytosis (ChAc) is caused by mutations in *hVPS13A* gene. The hVps13A protein interacts with actin and regulates the level of phosphatidylinositol 4-phosphate (PI4P) in the membranes of neuronal cells. Yeast Vps13 is involved in vacuolar protein transport and, like hVps13A, participates in PI4P metabolism. Vps13 proteins are conserved in eukaryotes, but their molecular function remains unknown. One of the mutations found in ChAc patients causes amino acids substitution I2771R which affects the localization of hVps13A in skeletal muscles. To dissect the mechanism of pathogenesis of I2771R, we created and analyzed a yeast strain carrying the equivalent mutation. Here we show that in yeast, substitution I2749R causes dysfunction of Vps13 protein in endocytosis and vacuolar transport, although the level of the protein is not affected, suggesting loss of function. We also show that Vps13, like hVps13A, influences actin cytoskeleton organization and binds actin in immunoprecipitation experiments. Vps13-I2749R binds actin, but does not function in the actin cytoskeleton organization. Moreover, we show that Vps13 binds phospholipids, especially phosphatidylinositol 3-phosphate (PI3P), via its SHR_BD and APT1 domains. Substitution I2749R attenuates this ability. Finally, the localization of Vps13-GFP is altered when cellular levels of PI3P are decreased indicating its trafficking within the endosomal membrane system. These results suggest that PI3P regulates the functioning of Vps13, both in protein trafficking and actin cytoskeleton organization. Attenuation of PI3P-binding ability in the mutant hVps13A protein may be one of the reasons for its mislocalization and disrupted function in cells of patients suffering from ChAc.

## Introduction

The Vps13 proteins are conserved in all eukaryotic organisms. In the human genome, Vps13 proteins are encoded by four genes *VPS13A*, *VPS13B*, *VPS13C* and *VPS13D* (1). For clarity *VPS13A* is further referred to as *hVPS13A.* Mutations in *hVPS13A* coding for the hVps13A protein, also named chorein, are the cause of a rare autosomal disorder, chorea-acanthocytosis (ChAc; OMIM 200150) (2), characterized by adult-onset chorea, progressive neurodegeneration and abnormal erythrocyte morphology - acanthocytosis. Most mutations found in ChAc patients lead to the generation of a premature stop codon and therefore cause a marked reduction of the mutant protein level in cells probably due a premature termination of translation and degradation of the *hVPS13A* transcript via nonsense mediated decay (3). However, several missense mutations have also been described. One of them, causes amino acid (aa) substitution I2771R and affects the localization of the hVps13A in skeletal muscles (4). The hVps13A is apparently involved in actin cytoskeleton organization and dynamics, since red blood cells and platelets from ChAc patients display depolymerization of the cortical actin cytoskeleton probably causing the acanthocytosis (5,6). Moreover an interaction between hVps13A and β-actin has been shown (7). The hVps13A protein, plays a role in metabolism of phospholipids, as it was shown that knockout of human *hVPS13A* led to a reduction in the level of phosphatidylinositol 4-phosphate (PI4P) in the Golgi apparatus and plasma membrane in the neuronal model PC12 cells (8). However, the molecular function of hVps13A is still unknown.

In the yeast *Saccharomyces cerevisiae*, which is a unicellular model eukaryotic organism, the single *VPS13* (*YLL040C*) gene codes for a protein which is involved in the transport of proteins from the Golgi apparatus to the vacuole, as documented by mislocalization of carboxypeptidase Y (CPY) (9–11), in the endocytic transport of lipid membranes from the plasma membrane to the vacuole, as was shown using FM4-64 lipophylic dye (12) and also in sporulation of diploids (11,13). Vps13 was localized in endosomes and prospore membrane (13,14). Vps13, like hVps13A, may also play a role in the regulation of the actin cytoskeleton and phospholipid metabolism, since it was captured in an *in vitro* actin assembly assay using microbeads coated with Las17, an activator of actin nucleation complex (15). In addition, the prospore membrane from *vps13Δ* strain has reduced phosphatidic acid (PA), PI4P and phosphatidylinositol 4,5-bisphosphate (PI(4,5)P_2_) content (13). Similarities in phenotypes caused by Vps13 protein dysfunction in cells from patients and in yeast make this organism a good model to study the effects of human Vps13A mutations on cellular functions.

The fact that Vps13 proteins influence the level of phosphoderivatives of phosphatidylinositol (PIPs) suggests that this defect might be the main problem in patients. It is well documented that PIPs are not only structural components of the lipid bilayers but also a key regulators of many cellular processes in eukaryotes including vesicular transport, cell proliferation, and actin cytoskeleton organization (16). Different phosphoinositide species are located in a specific cellular compartments and, hence, contribute to membrane identity (17). PIPs also serve as specific membrane-anchored determinants for the recruitment of a wide range of proteins, which can interact with these lipids via different conserved modular binding motifs. For example, PI4P, which is synthesized in yeast cells at the Golgi apparatus and at the plasma membrane, functions in multiple membrane trafficking pathways. PI4P production at the Golgi has a crucial role in maintaining secretory protein export to the cell surface, trafficking of cargoes to the vacuole, and in endocytic protein transport (18–20). The PI4P synthesized at the plasma membrane is implicated in regulation of endocytic trafficking mostly by regulation of actin cytoskeleton organization (21) and is a substrate for the synthesis of PI(4,5)P_2_ which regulates endocytosis, exocytosis, cytokinesis, maintenance of cell polarity, and actin cytoskeleton organization. PI(4,5)P_2_ functions not only as a localization determinant but also some effectors rely on PI(4,5)P_2_ binding to regulate their activities directly. For example, it accelerates the formation of actin networks during endocytic vesicle budding. More than 30 actin filament-binding proteins have been reported to associate directly with phosphoinositides (22). In contrast to PI4P and PI(4,5)P_2_, phosphatidylinositol 3-phosphate (PI3P) is located predominantly at the endosomal membrane, where it recruits different proteins, usually sharing a small subset of domains that interact directly with PI3P (17,23–25). The best characterized PI3P-binding domains are FYVE and PX (26). Two complexes: endosomal sorting complexes required for transport (ESCRTs) (25) and retromer complex (24) are examples of complexes recruited to endosomes based on PI3P binding. ESCRT complexes are required for the formation of the multivesicular body (also called late endosome), a crucial step in the delivery of cargo destined for degradation in the vacuole (27), while retromer complex is required for endosome-to-Golgi retrograde transport and recycling of proteins from the endosome to the plasma membrane (24). PI3P is also enriched in preautophagosomal structure and the isolation membrane, and is required for localization of Atg18-Atg2 complex of core autophagy proteins to these structures (28,29). Atg18 β-propeller protein binds PI3P via an FRRG motif (30,31) and Atg2 was recently found to contain an APT1 domain with the ability to bind PI3P independently of Atg18 (32). Atg2 proteins show some homology to Vps13 proteins (1) and therefore may play similar molecular functions in different processes.

To shed more light on how the I2771R substitution found in ChAc patient affects hVps13A, we constructed a yeast strain producing Vps13 with an equivalent substitution, *vps13-I2749R*, and studied its effects on protein trafficking. We tested the involvement of wild type and mutant versions of Vps13 in actin cytoskeleton organization and their ability to bind actin and lipids, and we discovered that Vps13 specifically binds PI3P while in Vps13-I2749R this binding is compromised. Additionally, the Vps13-I2749R-GFP was mislocalized in cells with decreased level of PI3P. Our results suggest that functioning of Vps13 is regulated by its interaction with PI3P, and losing PI3P-binding ability by hVps13-I2749R mutant protein accompanied with its abnormal cellular cycling might be a reason for its pathogenicity in some patients suffering for ChAc.

## Results

### Vps13-I2749R mutation, an equivalent of human mutation I2771R found in patient suffering from ChAc, impairs the function of the yeast Vps13 protein

**Figure 1:**
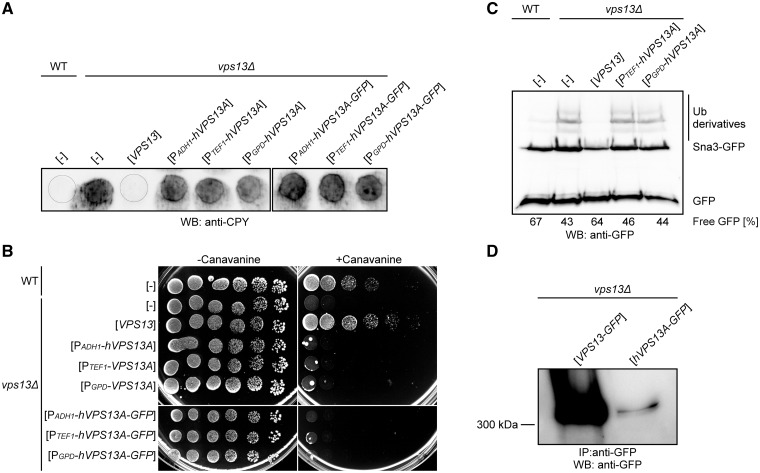
Human *hVPS13A* expressed in yeast cells does not complement *vps13Δ* defects*. ***(A**) Secretion of CPY. Yeast cultures were spotted on nitrocellulose membrane plated on SC-leu medium, incubated for 14-16 h and washed off. The level of secreted CPY was estimated using anti-CPY antibody. (**B**) Sensitivity of yeast strains to L-canavanine. Serial dilutions of wild type and *vps13Δ* strains bearing indicated plasmids were spotted on synthetic minimal medium supplemented with (3 µg ml ^−^ ^1^) or without L-canavanine. (**C**) Degradation of Sna3-GFP protein. Cells as in B were grown in SC-leu-ura medium to log-phase and total protein extracts were analyzed by SDS-PAGE followed by Sna3-GFP and GFP detection with anti-GFP antibody. Ubiquitinated (Ub) Sna3-GFP derivatives are indicated. The ratio GFP/(Sna3-GFP + Sna3-GFP-Ub + GFP) was calculated and is given for each strain as percentage of free GFP. (**D**) The detection of the hVps13A protein in yeast cells. Proteins extracted from *vps13Δ* strain expressing *VPS13-GFP* or *hVPS13A-GFP* under the *TEF1* promoter were immunoprecipitated, subjected to SDS-PAGE and western blot analysis with anti-GFP antibody.

Yeasts are widely used as a model organism to study human proteins and to improve the understanding of diverse human diseases (33–35). We therefore used the yeast cell system to study hVps13A function. First, we tested the ability of human *hVPS13A* to complement the defects of *vps13Δ* cells. The splice variant 1 of *hVPS13A* was used in this study as it is the most abundant one (1). The *vps13Δ* strain was transformed with single copy plasmids bearing *hVPS13A* or *hVPS13A-GFP* under the transcriptional control of three constitutive promoters of increasing strength: *ADH1*, *TEF1*, and *GPD* (*TDH3*) (36) and with plasmid bearing yeast *VPS13* with native promoter. To check the functionality of the human protein, complementation of the most characteristic phenotype of *vps13Δ* – missorting of CPY – was tested. CPY is a native vacuolar peptidase, but in *vps13Δ* mutant it is secreted from cells (12). Yeast cells were grown on nitrocellulose membrane on an SC-leu plate, washed out from the membrane surface and the secreted CPY was detected by western blotting using anti-CPY antibody. The *vps13Δ* strain bearing *hVPS13A* still transported CPY outside the cells, at a similar level as the *vps13Δ* control. This lack of complementation was independent of the strength of the promoter driving expression of *hVPS13A* (Fig. 1A). We also checked complementation of the other *vps13Δ* phenotypes related to protein trafficking: hypersensitivity to canavanine and degradation of the Sna3-GFP protein. Yeast cells lacking *VPS13* gene were more sensitive to canavanine, the toxic analog of arginine, which uses the same permease, Can1, to enter cells (Fig. 1B), indicating the defect of *vps13Δ* cells in Can1 endocytosis. Also the transport and degradation in the vacuole of the Sna3-GFP, an adapter protein involved in multivesicular body sorting of proteins and transport to the vacuole (37), is disturbed in this strain as assessed by accumulation of Sna3-GFP and Sna3-GFP ubiquitinated species and diminished ratio of the degradation product, GFP (Fig. 1C). All *vps13Δ* phenotypes tested were fully complemented by yeast *VPS13* but in contrast, none of them of was suppressed by the expression of the *hVPS13A* gene or *hVPS13-GFP* fusion (Fig. 1A, B and C). In other control experiments, we used plasmids bearing the *VPS13-GFP* gene under the control of *ADH1*, *TEF1* and *GPD* promoters and all these plasmids $132#?>suppressed the abnormal secretion of CPY and restored growth of *vps13Δ* strain on canavanine-containing medium (Supplementary Material, Fig. S1A and B). To find the reason for the lack of *vps13Δ* complementation, the cellular levels of hVps13A and yeast Vps13 proteins C-terminally tagged with GFP were compared using anti-GFP antibody. The hVps13A-GFP was not detectable in total cell extracts (not shown) but was observed in immunoprecipitates after using magnetic beads coated with anti-GFP antibody, indicating that the level of human protein in yeast is extremely low (Fig. 1D). This shows that *hVPS13A*-GFP, and possibly also untagged *hVPS13*, may be poorly expressed or be unstable and that may be a reason for lack of complementation of *vps13Δ* defects.

**Figure 2:**
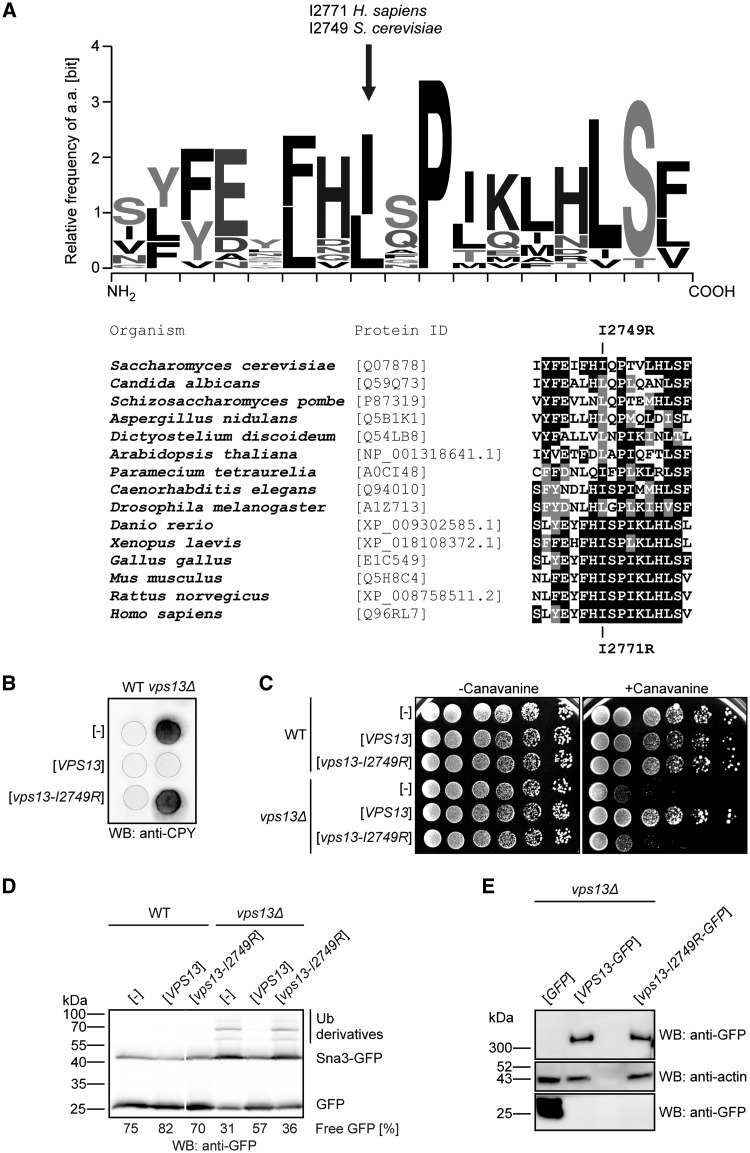
Yeast *VPS13* gene bearing the ChAc mutation I2749R does not complement deletion of *VPS13* in yeast cells. (**A**) The homology of Vps13 region containing the ChAc mutation (I2771R in human and I2749R in yeast). Logo diagram showing sequence conservation of the Vps13 orthologs from 15 model organisms was obtained using the WebLogo 3 program (67). Alignment was prepared using Clustal Omega tool (EMBL-EBI). (**B)** Secretion of CPY. Yeast cultures expressing wild type and mutant *VPS13* under control of *VPS13* native promoter were grown and analyzed as in Figure 1A. (**C**) Sensitivity of cells to L-canavanine. Serial dilutions of wild type and *vps13Δ* strains bearing plasmids containing wild type and mutant *VPS13* under the control of *VPS13* native promoter were spotted on medium supplemented with (1 µg ml ^−^ ^1^) or without L-canavanine. (**D**) Degradation of Sna3-GFP protein. Cells expressing P_*VPS13*_-*VPS13* or P_*VPS13*_-*vps13-I2749R* were grown in SC-leu-ura medium to log-phase and total protein extracts were analyzed by SDS-PAGE followed by Sna3-GFP detection with anti-GFP antibody. Irrelevant lane was removed. Percentage of free GFP [GFP/(Sna3-GFP + Sna3-GFP-Ub + GFP)] in each lane is given. (**E**) The level of Vps13-I2749R-GFP in yeast cells. Cells transformed with plasmids bearing P_*TEF1*_-*VPS13-GFP* or P_*TEF1*_-*vps13-I2749R-GFP* were grown to log-phase, total protein extracts were prepared and analyzed by SDS-PAGE followed by anti-GFP western blotting.

Thus, we introduced the human mutation found in ChAc patient in the yeast *VPS13* gene and tested the mutant for complementation of *vps13Δ* defects. The amino acid sequence of hVps13A (UniProtKB ID: Q96RL7-1) was compared with that of yeast Vps13 (SGD ID: S000003963) using the Clustal Omega tool (EMBL-EBI). We found that the I2771R substitution in ChAc patients (4) is present in the evolutionary conserved region of hVps13A and our multiple sequence analyses show that the hydrophobic nature of this position is conserved in all species tested (Fig. 2A). A mutation causing equivalent substitution, I2749R, was introduced into yeast *VPS13* gene under its native promoter and the ability to complement the *vps13Δ* defects was tested. In contrast to wild type *VPS13* gene the *vps13-I2749R* allele was unable to suppress *vps13Δ* defects, such as canavanine hypersensitivity, secretion of CPY and delay in Sna3-GFP degradation (Fig. 2B, C and D). This *vps13-I2749R* allele did not influence the wild type strain growth, canavanine sensitivity, Sna3-GFP degradation and CPY sorting indicating that mutation is recessive. To test if lack of complementation could be a result of decreased level of the mutant protein, western blot analysis of cell extracts derived from *vps13Δ* expressing wild type or mutant P*_TEF1_-VPS13-GFP* was performed. The level of mutant Vps13-I2749R-GFP protein was similar to wild type Vps13-GFP protein (Fig. 2E), showing that the reason for the non-functionality of the I2749R substitution is not due to a significant reduction in its level.

### Vps13-I2749R expressing cells have aberrant actin cytoskeleton organization

**Figure 3:**
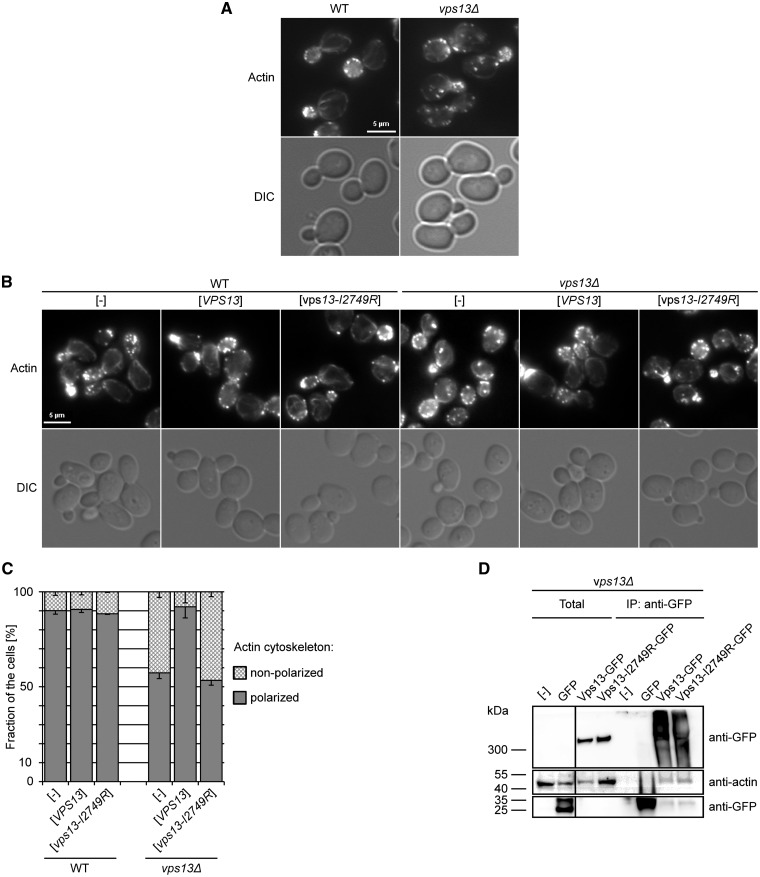
Vps13 influences the actin cytoskeleton organization and interacts with actin. (**A)** Organization of actin cytoskeleton in *vps13Δ* cells. Wild type and *vps13Δ* cells were grown to log-phase, fixed and stained using labeled phalloidine and observed by fluorescence microscopy. Scale bar, 5 µm. (**B).** Actin cytoskeleton organization in *vps13Δ* cells bearing P_*VPS13*_-*vps13-I2749R*. Assay was performed as in A. (**C)** Graphic representation of actin cytoskeleton organization in *vps13Δ* cells. At least 100 cells of each strain were observed and the percentage of cells with well-polarized and non-polarized actin cytoskeleton is indicated. Error bars represents standard deviations for three experiments. (**D)** Immunoprecipitation of wild type and mutant Vps13-GFP proteins. Yeast cells expressing P_*TEF1*_-*VPS13-GFP* or P_*TEF1*_-*VPS13-I2749R-GFP* were disrupted using glass beads and GFP-tagged proteins were precipitated using GFP-Trap magnetic beads. Samples were analyzed by standard SDS-PAGE followed by western blotting using anti-GFP and anti-actin antibodies. The 1/100 volume of extract taken to IP was loaded in Total lanes. Strain bearing empty plasmid or plasmid encoding *GFP* alone was used as a negative control. Irrelevant lane was removed.

Because of the protein transport defects and the unaffected level of Vps13-I2749R protein, we aimed to determine the reason for the loss of function phenotype of mutant expressing cells. It was shown previously that hVps13A is involved in actin cytoskeleton organization, since red blood cells and platelets from ChAc patients display depolymerization of the cortical actin cytoskeleton (5,6), and that hVps13A interacts with β-actin (7). Moreover, the yeast Vps13 protein was found among the components of Las17 activator-derived actin patch cortical network *in vitro* (15). The actin cytoskeleton is important for providing the forces required for a variety of cellular processes based on membrane dynamics, like endocytosis, exocytosis, and vesicular trafficking at the Golgi apparatus (38–40). Thus, we hypothesized that Vps13 plays a role in actin cytoskeleton organization also in yeast and that *vps13-I2749R* mutation might affect the interaction with actin network. To test this, staining of actin filaments using fluorescently labeled phalloidin was performed and the organization of the actin cytoskeleton in yeast *vps13Δ* cells was observed by fluorescence microscopy. In a wild type strain about 90% of dividing cells showed a well-organized actin cytoskeleton, the actin patches were concentrated in the bud and bud neck and actin cables were formed along the bud-mother cell axis, while in the *vps13Δ* mutant only about 50% of dividing cells exhibited normal polarization of the actin cytoskeleton. Among the cells with an abnormal actin cytoskeleton were those cells with more than five actin patches in the mother cell and with actin clumps in the central or distal part of the mother cell (Fig. 3A). Thus, Vps13 is involved in actin cytoskeleton organization in yeast as hVps13A is in human cells. Therefore, the organization of actin cytoskeleton in *vps13Δ* strain expressing *vps13-I2749R* from the plasmid was tested. *VPS13* and P_*TEF1*_-*VPS13-GFP* complemented fully the actin cytoskeleton defect of *vps13Δ* while *vps13-I2749R* expressing cells were still defective (Fig. 3B and C and Supplementary Material, Fig. S1). This result indicates that Vps13 is required for the normal organization of the actin cytoskeleton in yeast and the Vps13-I3749R is deficient also in this aspect of Vps13 functioning.

To further investigate the role of yeast Vps13 in actin cytoskeleton, the interaction between Vps13 and actin was tested. Vps13-GFP was immunoprecipitated from the total cell extracts of *vps13Δ* cells transformed with the plasmid bearing P*_TEF1-_VPS13-GFP* and the copurified proteins were analyzed for the presence of actin by western blotting. Indeed, actin was clearly visible in the immunoprecipitate, indicating that Vps13-GFP forms a complex with actin, directly or indirectly (Fig. 3D). Because the mutant *vps13-I2749R-GFP* failed to rescue all observed actin cytoskeleton and protein transport defects of *vps13Δ*, we asked if it was due to a loss of the Vps13 ability to interact with actin. To answer this question, the *vps13Δ* strain expressing *vps13-I2749R-GFP* was used for co-immunoprecipitation. Surprisingly, actin was present in the Vps13-I2749R immunoprecipitate similarly as for Vps13-GFP (Fig. 3D), indicating that the I2749R substitution does not compromise the interaction of Vps13 with actin but affects the actin cytoskeleton another way.

### Vps13 binds PI3P and I2749R substitution attenuates this interaction

**Figure 4:**
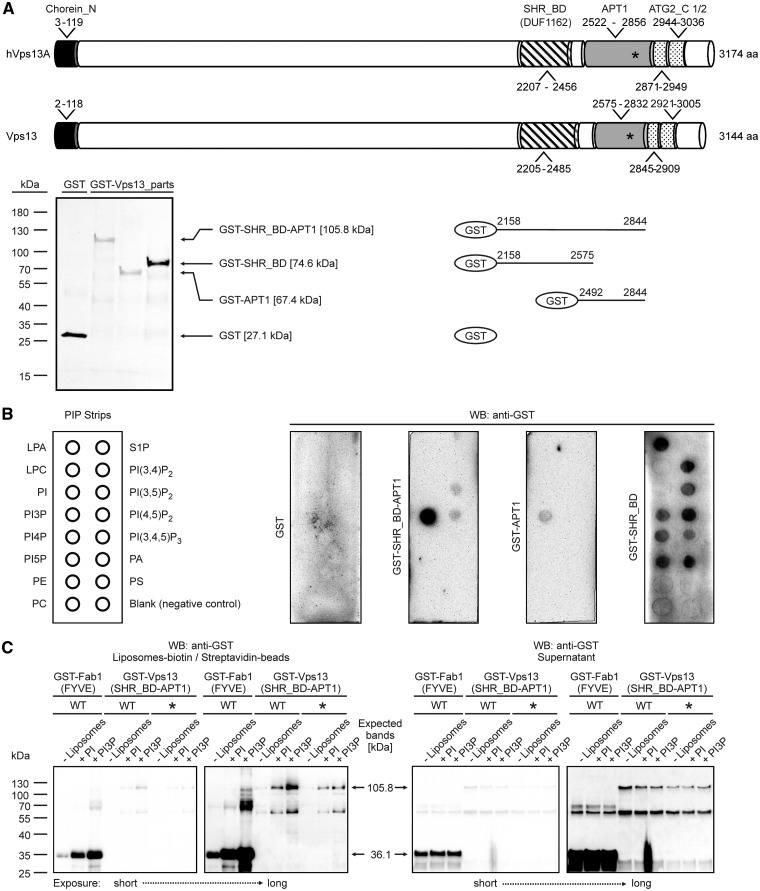
Vps13 fragments bind lipids and I2749R substitution attenuates this interaction. (**A**) Schematic representation of hVps13A and Vps13 domain structure and fragments used in PIP strip assay. Coomassie stained gel showing purified fragments used for PIP strip assay is also shown. Position of isoleucine (I) residue mutated to arginine (R) in ChAc patient and the corresponding site in yeast protein (I2771 in hVps13A and I2749 in yeast Vps13) is indicated by asterisks. Particular domains are indicated by boxes: black, chorein_N; striped, SHR_BD; grey, APT1; dotted, truncated and full-lengh ATG_C. (**B)** Binding of Vps13 fragments to lipids. GST-Vps13-(SHR_BD -APT1), GST-Vps13-(APT1), GST-Vps13-(SHR_BD) proteins were expressed in *E. coli* and purified. Up to 1 µg of purified proteins was used to test for binding to lipids deposited on the membrane (PIP strip). The fusion proteins were detected using anti-GST antibody. LPA, lysophosphatidic acid; LPC, lysophosphocholine; PI, phosphatidylinositol; PI3P, PI3-phosphate; PI4P, PI4-phosphate; PI5P, PI5-phosphate; PE, phosphatidylethanolamine; PC, phosphatidylcholine; S1P, sphingosine 1-phosphate; PI(3,4)P_2_, PI(3,4)-bisphosphate; PI(3,5)P_2_, PI(3,5)-bisphosphate; PI(4,5)P_2_, PI4,5-bisphosphate; PI(3,4,5)P_3_, PI(3,4,5)-trisphosphate; PA, phosphatidic acid; PS, phosphatidylserine. Experiment was performed at least two times with proteins from independent purifications. Representative result is shown. (**C)** Binding of Vps13 fragments to PI- and PI3P-containig liposomes. Crude extracts from *E. coli* containing wild type (WT) or I2749R mutant (*) variant of GST-Vps13-(SHR_BD -APT1) and GST-Fab1-(FYVE) as a positive control were incubated with indicated biotin-tagged liposomes. Liposomes were pull down using the streptavidin-covered beads and processes for western blot. All pull down fractions and 32 μl of each supernatant were loaded on the gel. The fusion proteins were detected using anti-GST antibody. Experiment was performed three times. Representative result is shown.

Because the interaction of Vps13 protein with actin is not affected by the I2749R substitution, we analyzed the structural organization of Vps13 to identify putative motifs that may bind other factors important for functioning of Vps13. The Vps13 protein contains two well-defined domains (Conserved Domains database, CDD NCBI): the N-terminal Chorein_N domain, which is proposed to form a leucine zipper for oligomerization or protein-protein interactions (41); and the SHR_BD (SHR-binding domain; previously known as DUF1162). SHR_BD is present in SHRUBBY (At5g24740), a vacuolar sorting protein of *A. thaliana* (42). The I2749R amino acid substitution was located in the region which was not previously characterized. Our bioinformatics analysis using the HHpred program (43; http://toolkit.lmb.uni-muenchen.de/hhpred; date last accessed February 8, 2017) additionally identified Vps13 region similar to the APT1 domain (Probab = 99.68%, E-value = 5.9e-15, Score = 141.23; PF10351, aa 2575-2832) (Supplementary Material, Fig. S2) and two ATG_C (Autophagy-related protein C terminal domain) domains – one full-length (aa 2921-3005) and one truncated (aa 2845-2909) (Fig. 4A). These domains (APT1, aa 2522-2856 and ATG_C, aa 2871-3036) were also found in hVps13A (Fig. 4A). The APT1 domain was first described in the Golgi apparatus protein APT1 of maize, involved in the growth of pollen tubes (44). We have recently found that APT1 domain is also present in yeast Atg2, a core autophagy protein, and we also show that APT1 of Atg2 binds PI3P (32). Since the I2749R substitution is located in the distal part of the putative APT1 domain of Vps13 (Fig. 4A), we speculated that APT1 of Vps13 can possibly also bind lipids and that this substitution may affect the lipid-binding ability. Since our analysis indicated that SHR_BD and APT1 may also be distantly related domains, we included SHR_BD in further analysis.

To test for lipid interaction, Vps13 fragments, containing the APT1, SHR_BD or both these domains (Fig. 4A) fused with a GST tag at the N terminus, were produced in *E. coli.* Purified proteins were incubated with commercial PIP strips and the lipid-bound proteins were detected using anti-GST antibody. All three Vps13 fragments were able to interact with lipids, but with different specificity. SHR_BD-APT1 and APT1 domain alone bound almost exclusively PI3P and the binding was stronger for the longer fragment (Fig. 4B). The fragment containing the SHR_BD domain alone interacted equally well with all phosphoinositides and also with phosphatidic acid and lysophosphatidic acid (Fig. 4B). Although the PIP strip assay is qualitative rather than quantitative, the obtained results suggest that the SHR_BD domain is responsible for non-specific phospholipid binding while the APT1 domains confers specificity on this interaction.

To compare lipid binding ability of wild type SHR_BD-APT1 fragment of Vps13 with its mutant version containing I2749R substitution, the liposome pull-down method was used. Total bacterial extracts containing GST-Vps13-(SHR_BD-APT1), GST-Vps13-I2749R-(SHR_BD-APT1) or GST-Fab1-(FYVE), a control fusion containing PI3P-binding domain of yeast PI3P 5-kinase Fab1 (45), were incubated with liposomes containing PI or PI3P. We observed that similarly to control GST-Fab1-(FYVE) protein, the wild type fragment of Vps13 was dominantly associated with PI3P-containing liposomes compared to PI-containing liposomes (Fig. 4C), (4.2% PI; 7% PI3P and 2.7% PI; 10% PI3P, respectively). Mutant Vps13 fragment associated more efficiently with PI and less efficiently with PI3P (4.5% PI; 5.4% PI3P) when compared to wild type fragment. These results suggest lower-binding ability to PI3P of mutant Vps13 SHR_BD-APT1 when amino acid I2749 is substituted for R. These results suggest further that the attenuation of Vps13 ability to bind PI3P lipid is sufficient to compromise its functioning in both, actin cytoskeleton organization and transport of CPY, Can1 and Sna3 proteins.

### The binding of Vps13 to PI3P regulates its localization

**Figure 5:**
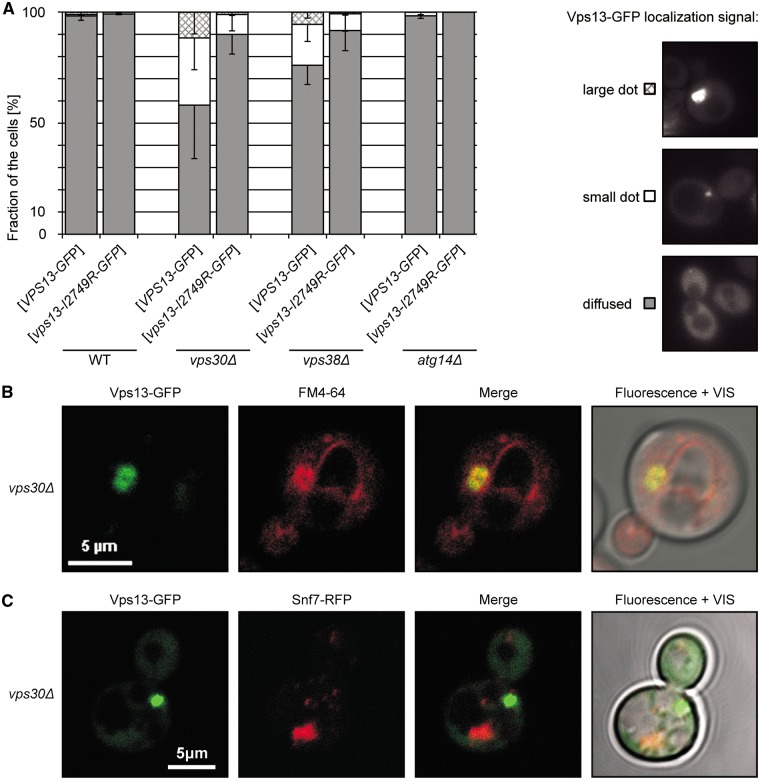
The level of PI3P influences the localization of Vps13-GFP. (**A**) Localization of Vps13-GFP in PI3P deficient *vps30Δ* and *vps38Δ* cells. Strains transformed with plasmid bearing P_*TEF1*_-*VPS13-GFP* were grown in SC-leu minimal medium to log-phase and the localization of Vps13-GFP was observed by fluorescence microscopy. At least 100 cells for each strain were observed. The percentage of cells displaying various Vps13-GFP distribution patterns, illustrated in the right-hand panel, is shown. (**B**) Colocalization of Vps13-GFP and FM4-64 in *vps30Δ* strain. The *vps30Δ* strain was transformed with a plasmid encoding P_*TEF1*_-*VPS13-GFP*. Cells were grown in SC-leu medium to log-phase and the localization of Vps13-GFP protein was observed by confocal microscopy. FM4-64 was used to visualize the compartments of endocytic pathway. Scale bar, 5 µm. (**C)** Localization of Vps13-GFP and Snf7-RFP in *vps30Δ* strain. The *SNF7-RFP vps30Δ* strain was transformed with a plasmid encoding P_*TEF1*_-*VPS13-GFP*. Cells were grown in SC-leu medium to log-phase and the localization of Snf7-RFP and Vps13-GFP proteins was observed by confocal microscopy.

Our results have indicated that binding to PI3P is important for functioning of Vps13. PI3P, similar to other phosphoderivatives of PI, is characteristic for some cellular compartments, such as endosomes and autophagosomes, and can confer membrane identity (17). It also recruits many proteins to its site of action, thus determining their localization and functioning in cellular processes. To determine whether PI3P influences the localization of Vps13, strains devoid of individual subunits of PI3-kinase expressing P_*TEF1*_-*VPS13-GFP* or P*_TEF1_-vps13-I2749R-GFP* from the plasmid were used. There is only one PI3-kinase in yeast which is the sole source of PI3P and phosphorylates only PI. It is part of two distinct complexes which function in separate membrane trafficking processes. Complex I plays an essential role in autophagy and is composed of the catalytic dimer Vps34-Vps15, and Vps30 and Atg14 subunits, while complex II functions in endosomal retrograde protein sorting, multivesicular body protein sorting and Golgi to vacuole transport, and instead of Atg14 contains Vps38 (17,46,47). In *vps30Δ* and *vps38Δ* mutants the level of PI3P is decreased about threefold and endosomal retrograde transport is defective while in *atg14Δ* the PI3-kinase complex I is not recruited to the phagophore assembly site and only autophagy is compromised (24,46). In wild type strains, Vps13-GFP was observed to be mainly diffuse in the cytoplasm though in a small proportion of cells small dots were observed, possibly endosomes. In contrast, in *vps30Δ* strain, Vps13-GFP formed a dot adjacent to vacuole in 40% of cells and this dot was very large in 10% of cells. This increased punctate appearance of Vps13-GFP was similar in *vps38Δ* strain and was absent in *atg14Δ* strain (Fig. 5A), thus it correlates with decreased PI3P level. The observed large dots of Vps13-GFP were located perivacuolarly and colocalized with the membrane compartment stained by FM4-64 lipophylic dye which is taken up to the cell by endocytosis (Fig. 5B), suggesting that Vps13-GFP excessively accumulates on the surface of the membraneous structure of endocytic origin, likely endosome or multivesicular body, when the endosomal level of PI3P is low. To test that prediction, Snf7-RFP, an ESCRT-3 protein, was used as a marker of the multivesicular body/late endosome (14). Surprisingly, Vps13-GFP and Snf7-RFP did not colocalize, they accumulated in different perivacuolar spots in the cell (Fig. 5C) and is not clear if Snf7-RFP is endosomal in *vps30Δ* mutant cells. In contrast, Vps13-I2749R-GFP was found in small dots only in 10% of *vps30Δ* and *vps38Δ* cells and a large dot was not observed in these cells (Fig. 5A) indicating that I2749R mutation prevents endosomal accumulation of Vps13 or formation of this compartment in this condition. This Vps13-GFP endosomal localization together with PI3P-binding ability support the idea that Vps13 may cycle between cytoplasmic and endosomal localization and this cycling, which depends on PI3P binding, is required for endosomal sorting and actin cytoskeleton organization, and is compromised by I2749R substitution.

## Discussion

Despite the fact that *hVPS13A* is involved in the human disease chorea-acanthocytosis, little is known about its structure and molecular function. Here, we attempted to broaden knowledge about the role of Vps13 in cells and find how mutations can affect its function in yeast. The present study describes the construction and properties of the yeast strain producing mutant Vps13 with changes equivalent to the I2771R substitution in hVps13A found in ChAc patients. This replacement of the conserved hydrophobic isoleucine residue by positively charged arginine within the putative APT1 domain is detrimental to human cells, however how this pathogenic change affects hVps13A remained unknown. Yeast Vps13 is similar to human Vps13A, especially in the region of hVps13A modified by I2771R. Isoleucine 2771 is conserved in yeast, human and 64% of Vps13 sequences analyzed and in other is substituted by similar aa, leucine, suggesting that its pathogenic mutation to arginine may similarly affect both the human and yeast proteins. Here, we found new phenotypes of *vps13Δ* mutants and identified a novel APT1 domain in Vps13 and hVps13A proteins in which I2749/2771 is located, respectively. We also documented that this APT1 domain along with the preceding SHR_BD domain together, and independently, bind phosphorylated phosphatidyl-inositol lipids. Moreover, we show that I2749R substitution attenuates lipid binding ability of SHR_BD-APT1 fragment of Vps13 and results in Vps13 loss of function with an impact on actin cytoskeleton organization and vesicular transport.

To broaden the knowledge on *vps13Δ* mutants, various markers of vesicular traffic were tested and we found that cells devoid of Vps13 are defective in endocytosis of Can1, and Golgi to vacuole transport and degradation of Sna3-GFP. We included these new phenotypes for further complementation analysis in addition to the classical test for CPY secretion, the first *vps13Δ* phenotype described (9–11). Our other finding that yeast *vps13Δ* mutants show defects in actin cytoskeleton organization and Vps13 is present with actin in immunoprecipitates similarly to hVps13A, also adds further support to the use of yeast as a suitable model organism to study effects of hVps13A mutations. The actin cytoskeleton is implicated in several vesicular transport steps, such as plasma membrane endocytic invagination and endosome movement (48–51), multivesicular body biogenesis (50,52) and retrograde transport (53) in yeast and mammals. Various sets of proteins are involved in building actin filaments networks in these different locations (54) and bridge actin cytoskeleton with membrane dynamics. Actin cytoskeleton defects correlate well with vesicular transport defects in *vps13Δ* but we could not discriminate if Vps13 primary function is in actin cytoskeleton or membrane dynamics or it is a bridging protein.

Complementary to our findings, Lang and coworkers have recently shown that Vps13 is localized at membrane contact sites (MCS) in yeast (55). MCS are structures where two membranes are tethered in close apposition but not fused. These junctions allow the exchange of ions, metabolites and lipids between organelles in response to a cell’s needs (56). Vps13^GFP, with internal GFP, was found in endosomes and in MCS integrating the mitochondria and vacuole (called vCLAMP) when cells were grown on glucose medium, but in nuclear-vacuolar junctions when shifted to glycerol medium (55). These findings were further confirmed and extended by documenting that *vps13* mutants have defects in mitochondrial functioning and Vps13 protein, besides vCLAMP and nuclear-vacuolar junctions, is also located in endosome-mitochondrial junctions and through these different localizations promotes different processes (57). It was demonstrated that different processes known to require functional Vps13: sporulation, transport of CPY to the vacuole and the formation of some MCS are genetically independent and can be separated by different *VPS13* mutations (57). Thus, it is possible that Vps13 carries out several independent functions – in actin cytoskeleton organization, endosomal trafficking, sporulation, and in MCS formation or carries one function in these different processes but requires different domains to act in these various locations. Many of the proteins being important for the formation and function of MCS have been linked to other cellular processes (58–61). Thus, function of Vps13 in MCS may be linked to its ability to regulate the actin cytoskeleton organization and protein transport at the same time.

Beyond actin-binding ability, we have found that SHR_BD and APT1 domains of Vps13 interact with PIPs together and independently. SHR_BD domain bound many of the PIPs tested while the APT1 domain conferred specificity towards PI3P. The SHR_BD domain is also presented in hVps13A and we have also found a putative APT1 domain in this protein, suggesting that hVps13A may also interact with specific phospholipids. These findings added Vps13 family proteins to the superfamily of lipid-binding proteins and supports the notion that Vps13 could be able to bind membranes, including endosomal membranes enriched in PI3P, and even tether two membranes together in various MCSs via two lipid-binding domains. While this work was in revision, data complementary to our data were published which show that Vps13 contains at least two more lipid-binding domains at the N- and C-terminus, respectively (62). Moreover, the authors predicted the presence of PI3P-binding domain in the central part of the Vps13 protein (62) and that is in agreement with our findings.

PI3P seems also to regulate Vps13 localization, since we have observed that a decreased level of endosomal PI3P in *vps30Δ* cells markedly altered the localization of the Vps13 protein; it was accumulated in a large perivacuolar membrane compartment of endocytic origin reminiscent to the class E compartment observed when vacuolar sorting is blocked (63) but such a compartment is normally not observed in *vps30Δ* (64). This compartment may also be an insoluble protein deposit where abnormal, aggregated or overproduced proteins are sequestered (32,65) and where Vps13-GFP may be held when endocytic traffic is disturbed. It was observed previously that decreased level of PI3P leads to diffusion of Vps5-GFP and Vps17-GFP, subunits of the retromer complex, and GFP-Vps27, an ESCRT-0 protein, from the endosome to cytoplasm (24,25). Vps13 behaved in the opposite way, as PI3P would be required for its release from endosome. However, in contrast to our data showing that *VPS13-GFP* is fully functional in the actin cytoskeleton and vesicular transport, recent reports indicate that Vps13-GFP (tagged at the C terminus) is inactive in MCS formation (55,57), thus we cannot exclude that the Vps13-GFP localization, we observed in mutants, at least in part results from this inability.

Modeling of I2771R substitution found in APT1 domain of hVps13A in ChAc patient (I2749R in yeast) showed that it greatly affects its function. Despite the similar level of mutant Vps13-I2749R-GFP to that of wild type one, it was unable to complement *vps13Δ* defects, such as depolarization of actin cytoskeleton, secretion of CPY, hypersensitivity to canavanine and delay in Sna3-GFP degradation, indicating a loss of function. This mutant phenotype was linked with diminished lipid-binding ability, specifically to PI3P, and a change in endosomal localization when the endosomal level of PI3P is low. Another four missense mutations found in ChAc patients were recently modeled in yeast cells and respective mutants, *vps13-L66P*, *vps13-C89K*, *vps13-L1107P* and *VPS13-Y2702C*, were tested for sporulation, CPY sorting and synthetic lethality with *mmm1Δ* (57), an ERMES mutant (66). *VPS13-Y2702C* did not show defects of these processes, all others show efficient sporulation but were synthetically lethal with *mmm1*Δ allele. The *vps13-L1107P* mutant additionally displayed a CPY sorting defect (57). None of these mutation caused amino acid substitutions in the APT1 domain of Vps13. Thus, our analysis is complementary to these findings and shows that for proper transport of CPY, in addition to leucine 1107, isoleucine 2749 in the APT1 domain is required. Therefore, two distinct domains of Vps13 must cooperate for this one function. Our recent analysis shows that *vps13-I2749R* mutation also causes defect in sporulation and lethality with *mmm1Δ* (Supplementary Material, Tables S1 and S2) showing again for cooperation of several domains in one process. Based on our and other results, we hypothesize that the I2771R mutation may alter PI3P binding by APT1 of hVps13A and the interaction of hVps13A with PI3P-enriched membranes, and this interferes with hVps13A trafficking and formation of MCS. That leads to the observed transport and actin cytoskeleton defects in human cells and could be one of the mechanisms of ChAc pathogenesis.

Our and other studies show that it is possible in yeast to successfully mimic *hVPS13A* mutations found in ChAc patients and this model can provide us useful information about function of Vps13 protein and possible mechanism of the disease. Yeast ChAc model is also potentially useful for high-throughput chemical screen to select possible drugs.

## Materials and Methods

### Strains, media and growth conditions

*E. coli* strain DH5α was used for plasmid propagation. The yeast *S. cerevisiae* strains were used in this study: BY4741 (*MAT***a***his3Δ1 leu2Δ0 met15Δ0 ura3Δ0)*, BY4741 *vps13Δ (MAT***a***his3Δ1 leu2Δ0, met15Δ0 ura3Δ0 vps13Δ::KanMX)*, BY4741 *vps30Δ (MAT***a***his3Δ1 leu2Δ0, met15Δ0 ura3Δ0 vps30Δ::KanMX)*, BY4741 *vps38Δ (MAT***a***his3Δ1 leu2Δ0, met15Δ0 ura3Δ0 vps38Δ::KanMX)*, BY4741 *atg14Δ (MAT***a***his3Δ1 leu2Δ0, met15Δ0 ura3Δ0 atg14Δ::KanMX)* (Open Biosystem) and KAY687 (*MAT*α *SNF7-RFP::KanMX his3Δ1 leu2Δ0 lys2Δ0 ura3Δ0*) (14).

To construct strain *SNF7-RFP vps30Δ*, first *KanMX* at *SNF7* locus was replaced by *HIS3* in KAY687 and next *vps30Δ::KanMX* casette was introduced and the resulting strain was named KJK173.

Yeast cells were grown at 28 °C in liquid YPD medium (1% yeast extract, 2% bactopeptone, 2% glucose) or in synthetic complete medium SC (0.67% yeast nitrogen base without amino acids, 2% glucose, 0.2% complete supplement mixture -ade-his-leu-trp-ura) with desired supplements (uracil and amino acids) if selection for plasmid maintenance was required. For growth test, the cells were grown overnight in SC-leu medium, pelleted and resuspended to OD_600 _=_ _1 in synthetic minimal medium SD (0.67% yeast nitrogen base without amino acids, 2% glucose) with required supplements and 1:4 serial dilutions were prepared. Aliquots of each dilution were spotted on SD-leu minimal plates with required supplements and SD-leu-arg supplemented with 1-3 µg ml ^−^ ^1^or without L-canavanine and then incubated at 28 °C for 4 days.

### Plasmids and plasmid construction

Plasmids used in this study are listed in Table 1. Plasmid pRS415-VPS13 was constructed by gap-repair in yeast cells and contains *VPS13* open reading frame (ORF) under the transcriptional control of native promoter and terminator sequences (chromosome XII coordinates: 64064 - 53920). For C-terminal GFP tagging of Vps13, the pUG35-VPS13 plasmids was produced by amplification of two fragments of the *VPS13* ORF by PCR using pRS415-VPS13 as a template and primers providing restriction sites (a ∼700-bp 5’-end fragment flanked by XmaI and EcoRI sites and the ∼400-bp 3’-end one flanked by EcoRI and XhoI sites and devoid of the STOP codon), ligation of both fragments into XmaI/XhoI-linearized pUG35 vector and subsequent transfer of the 8361-bp BclI-BclI central part of *VPS13* from pRS415-VPS13. The orientation of the BclI-BclI fragment was checked by restriction analysis. Plasmids p415-P_ADH1_-VPS13-GFP, p415-P_TEF1_-VPS13-GFP and p415-P_GPD_-VPS13-GFP were obtained by subcloning of the *VPS13-GFP* coding sequence with the *CYC1* terminator as a Cfr9I-Eco52I fragment from pUG35-VPS13 into p415-P_ADH1,_ -P_TEF1_ and -P_GPD_ vectors (36).

**Table 1:** Plasmids used in this study

Plasmid	Source or reference
p415-P_ADH1_	(36)
p415-P_ADH1_-hVPS13A	This study
p415-P_ADH1_-hVPS13A-GFP	This study
p415-P_ADH1_-VPS13-GFP	This study
p415-P_GPD_	(36)
p415-P_GPD_-hVPS13A	This study
p415-P_GPD_-hVPS13A-GFP	This study
p415-P_GPD_-VPS13-GFP	This study
p415-P_TEF1_	(36)
p415-P_TEF1_-hVPS13A	This study
p415-P_TEF1_-hVPS13A-GFP	This study
p415-P_TEF1_-VPS13-GFP	This study
p415-P_TEF1_-vps13-I2749R-GFP	This study
p416-SNA3-GFP	(37)
pBluescript SKII	Agilent Technologies, Santa Clara, CA, USA
pBluescript-vps13-I2749R	This study
pDK106 (P_*tac*_-GST•tag-Thrombin_c.s.-TEV_c.s.- *fab1-(S233-D303)*)	D. Kolakowski, IBB PAS
pKF463 (P_*tac*_-GST•tag-Thrombin_c.s.-TEV_c.s.- 2×STOP)	(32)
pKF482 (P_*tac*_-GST•tag-Thrombin_c.s.-TEV_c.s.- *vps13-(S2492-P2844)*)	This study
pKF483 (P_*tac*_-GST•tag-Thrombin_c.s.-TEV_c.s.- *vps13-(S2158-P2844)*)	This study
pKF490 (P_*tac*_-GST•tag-Thrombin_c.s.-TEV_c.s.- *vps13-(S2158-K2575)*)	This study
pKF492 (P_*tac*_-GST•tag-Thrombin_c.s.-TEV_c.s.- *vps13-(S2158-P2844)-I2749R*)	This study
pRS415	(68)
pRS415-VPS13	This study
pRS415-vps13-I2749RC	This study
pUG35	J.H. Hegemann, University of Düsseldorf
pUG35-hVPS13A	This study
pUG35-VPS13	This study

All plasmids confer resistance to 100 µg ml ^−^ ^1^ ampicillin; c.s – cleavage site.

The I2749R (Att > Aga) mutation (numbering according to yeast Vps13 aa sequence, corresponds to human hVps13A position 2771) was introduced by site directed mutagenesis into *VPS13* fragment (bp 9221 - 8656) which was PCR-amplified with flanking PstI and HindIII restriction sites and cloned into pBluescript SKII giving pBluescript-vps13-I2749R. Two silent mutations, E2745E (GAg > GAa) and F2747F (TTc > TTt), were also introduced to generate additional PdmI and AsuII sites and to destroy one of the BglII sites, enabling simple confirmation of the presence of the mutated region by restriction analysis. The mutations were subsequently introduced into full-length *VPS13* by substitution of the 1545-bp BbvCI-AjiI DNA fragment of pRS415-VPS13 or p415-P_TEF1_-VPS13-GFP with the corresponding one from pBluescript-vps13-I2749R.

The human *hVPS13A* ORF was PCR-amplified without the STOP codon using cDNA (synthesized on mRNA isolated from human blood cells) as a template and primers with flanking Cfr9I and SalI restriction sites. The obtained product was digested with Cfr9I and SalI and ligated into Cfr9I/SalI-linearized pUG35. A fragment containing *hVPS13A*-*GFP*-T_*CYC1*_ was excised with Cfr9I and Eco52I from pUG35-hVPS13A plasmid and subcloned into p415-P_ADH1_, -P_TEF1_ and -P_GPD_ vectors in the same sites.

For production of truncated Vps13 variants in *E. coli*, appropriate constructs encoding N-terminally GST-tagged proteins were created. The pKF463 vector, encoding the GST tag alone, was used as a control. It is based on the pGEX-4T-1 backbone and carries a STOP codon directly downstream of the encoded TEV protease cleavage site (32). Appropriate *VPS13* fragments were PCR-amplified on genomic DNA of the S288C strain and cloned into pKF463. The *vps13-APT1* (aa S2492-P2844) and *vps13-SHR_BD -APT1* (aa S2158-P2844) fragments encoding the APT1 and the SHR_BD-APT1 domains were cloned, respectively, as 1077-bp or 2079-bp Esp3I-Esp3I fragments into 4978-bp AarI/SalI- or 4972-bp AarI/XhoI-digested pKF463 yielding pKF482 and pKF483, respectively. The SHR_BD-coding sequence was cloned as a 1308-bp Esp3I-Esp3I fragment (TEV_c.s.-*vps13_(S2158-K2575)_*-2 × STOP) into 4954-bp BamHI/XhoI-digested pKF463 giving pKF490. The I2749R mutation was introduced into *vps13-SHR_BD-APT1* sequence by substitution of the 677-bp NdeI-AjiI fragment of pKF483 with the equivalent one from pBluescript-vps13-I2749R.

Plasmid pDK106 encoding GST-Fab1-(FYVE) contains fragment of yeast *FAB1* gene encoding FYVE domain (aa S233-D303) on the pKF463 backbone.

### Western blot analysis

Yeast cells were grown at 28°C on SC-leu or SC-ura-leu to the log-phase. Protein extracts were prepared after disrupting cells with glass beads in 2x electrophoresis sample buffer (120 mM Tris-HCl pH 6.8, 2% SDS, 20% glycerol, 0.04% bromophenol blue, 10% β-mercaptoethanol) or in IP buffer (50 mM Tris-HCl pH 8.0, 0.2 mM CaCl_2_, 150 mM NaCl, 2% Triton X-100, 1 mM PMSF, Inhibitor protease cocktail (Sigma-Aldrich, Saint Louis, MI, USA)). Samples were analyzed by standard SDS-PAGE followed by western blotting using mouse monoclonal anti-GFP (Roche, Basel, Switzerland) or anti-actin (Millipore, Darmstadt, Germany) antibodies; secondary anti-mouse IgG horseradish peroxidase (HRP)-conjugated antibody (Dako, Glostrup, Denmark) and signal detection by enhanced chemiluminescence (Millipore).

### Secretion of CPY

To test the secretion of CPY the cells were grown overnight at 28 °C and then diluted to OD_600 _=_ _1. Ten microliters of each culture was dropped on nitrocellulose membrane on an SC-leu plate and incubated for 14–16 h at 28 °C. Then cells were washed off the membrane and the secreted CPY was detected with an anti-CPY antibody (Thermo Fisher Scientific, Waltham, MA, USA).

### Immunoprecipitation

Immunoprecipitation was performed using GFP-Trap (ChromoTek, Martinsried, Germany) according to the manufacturer protocol. The *vps13Δ* strains bearing empty plasmid or plasmids carrying *GFP*, *VPS13-GFP* or *vps13-I2749R-GFP* under the *TEF1* promoter were grown in SC-leu medium to log-phase. About 50 OD_600_ of culture was collected and cells were disrupted using glass beads in CoIP buffer (50 mM HEPES, pH 7.5, 100 mM NaCl, 1 mM MgCl_2,_ 0.5% Triton X-100, 1 mM PMSF; Inhibitor protease cocktail (Sigma-Aldrich)). To remove unbroken cells and cell debris, samples were centrifuged at 10 000 × g. The supernatant was incubated with GFP-Trap (ChromoTek) for 2 h at 4 °C. After incubation beads were washed five times with 50 mM HEPES pH 7.5. The samples were analyzed by SDS-PAGE followed by western blotting using mouse monoclonal anti-GFP (Roche) and mouse monoclonal anti-actin (Millipore) antibodies.

### Fluorescence and confocal microscopy

To observe membranes, yeast cells were grown to log-phase in SC-leu medium, collected, resuspended in ice-cold YPD medium supplemented with 40 µM FM4-64 (Thermo Fisher Scientific), and incubated for 30 min on ice. The cells were then washed with ice-cold YPD medium, incubated for 60 min at 30 °C and then the sodium azide and fluoride were added.

For actin cytoskeleton staining, cells were grown to log-phase, fixed for 2 h by the addition of formaldehyde to 3.7%, stained with 546 Alexa Fluor-conjugated phalloidin (Thermo Fisher Scientific) and washed.

To analyze localization of Vps13-GFP or Vps13-I2749R-GFP, the wild type, *vps30Δ, vps38Δ, atg14Δ* or *SNF7-RFP vps30Δ* cells were transformed with respective plasmids, grown, and were observed by fluorescence or confocal microscopy, as indicated.

Cells were viewed with an Eclipse E800 fluorescence microscope (Nikon, Tokyo, Japan) equipped with a DS-5Mc camera (Nikon). Images were collected using Lucia General 5.1 software (Laboratory Imaging Ltd., Praha, Czech Republic). The same fields were viewed by differential interference contrast (DIC) optics. Confocal imaging was perform using confocal laser scanning microscope EZ-C1 Eclipse TE2000-E (Nikon) equipped with a Plan Apo 60× objective (NA 1.4). Images were collected with EZ-C1 confocal V. 3.6 program (Nikon). Images were processed with EC1 Viewer 3.6 and Adobe Photoshop 8.0.

### Purification of Vps13 fragments and determination of their binding to lipids

Truncated variants of Vps13 were expressed as N-terminally GST-tagged recombinant proteins in *E. coli* BL21(DE3) strain propagated at 28 °C in 2 × LB medium supplemented with 50 mM HEPES pH 7.4 and 100 µg ml ^−^ ^1^ carbenicillin (Sigma-Aldrich) for plasmid maintenance. Expression was induced with 0.2 mM IPTG for 4-6 h. Then cells were pelleted, resuspended in phosphate-buffered saline (PBS) supplemented with protease inhibitor cocktail (complete Mini, EDTA-free; Roche) and lysed by sonication. The homogenate was clarified by incubation with benzonase endonuclease at 5-10 U ml ^−^ ^1^ (Merck Millipore) followed by centrifugation at 20 000 × g for 15 min at 4 °C. The supernatant was supplemented with 2% Triton X-100, 1 mM EDTA and 5 mM DL-dithiothreitol (DTT), and incubated with glutathione magnetic beads (Thermo Scientific) for 2-3 h at 4 °C. After washing steps, the bound proteins were eluted with 25 mM reduced glutathione in elution buffer (100 mM Tris·Cl pH 8.0, 150 mM NaCl, 2 mM EDTA, 2 mM DTT, 0.1% Tween-20) and the purity of eluted proteins was analyzed by SDS-PAGE.

Identification of lipids interacting with a given protein was done by an overlay assay. A membrane containing lipids (PIP Strips; Echelon Biosciences Inc., Salt Lake City, UT, USA) was blocked with 3% fatty acid-free BSA (Sigma-Aldrich) in PBS-T (PBS, 0.1% Tween-20) and then incubated overnight at 4 °C in blocking buffer with 0.5-1 µg ml ^−^ ^1^ of particular protein or GST alone as a negative control. Bound proteins were detected with anti-GST HRP-conjugated antibodies (Sigma-Aldrich).

### Liposome-binding assays

Liposomal assays were carried out using synthetic biotin-tagged PolyPIPosomes containing 5% (w/w) individual phosphoinositides (Echelon Biosciences Inc.). Crude *E. coli* extracts containing GST-Vps13-(SHR_BD-APT1), GST-Vps13-I2749R-(SHR_BD-APT1) or GST-Fab1-(FYVE) domain, as a positive control, were incubated with 10 µl of liposome mixtures in 1 ml reaction buffer (50 mM HEPES, pH 7.5, 100 mM NaCl, and 1 mM MgCl_2_) for 20 min at room temperature. Next, 50 μl of Dynabeads MyOne Streptavidin T1 (Invitrogen) were added to pull down the lipid–protein mixtures for 1 h at 4 °C. Following 5 washes with 0.5 ml reaction buffer, the beads were mixed with 2× sample buffer and processed for western blotting using anti-GST antibody.

## Supplementary Material

Supplementary Material is available at *HMG* online.

## Supplementary Material

Supplementary Data
