# Supplementary material for: Amino acid substitution equivalent to human chorea-acanthocytosis I2771R in yeast Vps13 protein affects its binding to phosphatidylinositol 3-phosphate
Source: Hum Mol Genet. 2017 Mar 1;26(8):1497–510. doi: 10.1093/hmg/ddx054 (PMC5393151; doi:10.1093/hmg/ddx054)
Supplement: Supplementary Data [file ddx054_Supp.pdf]

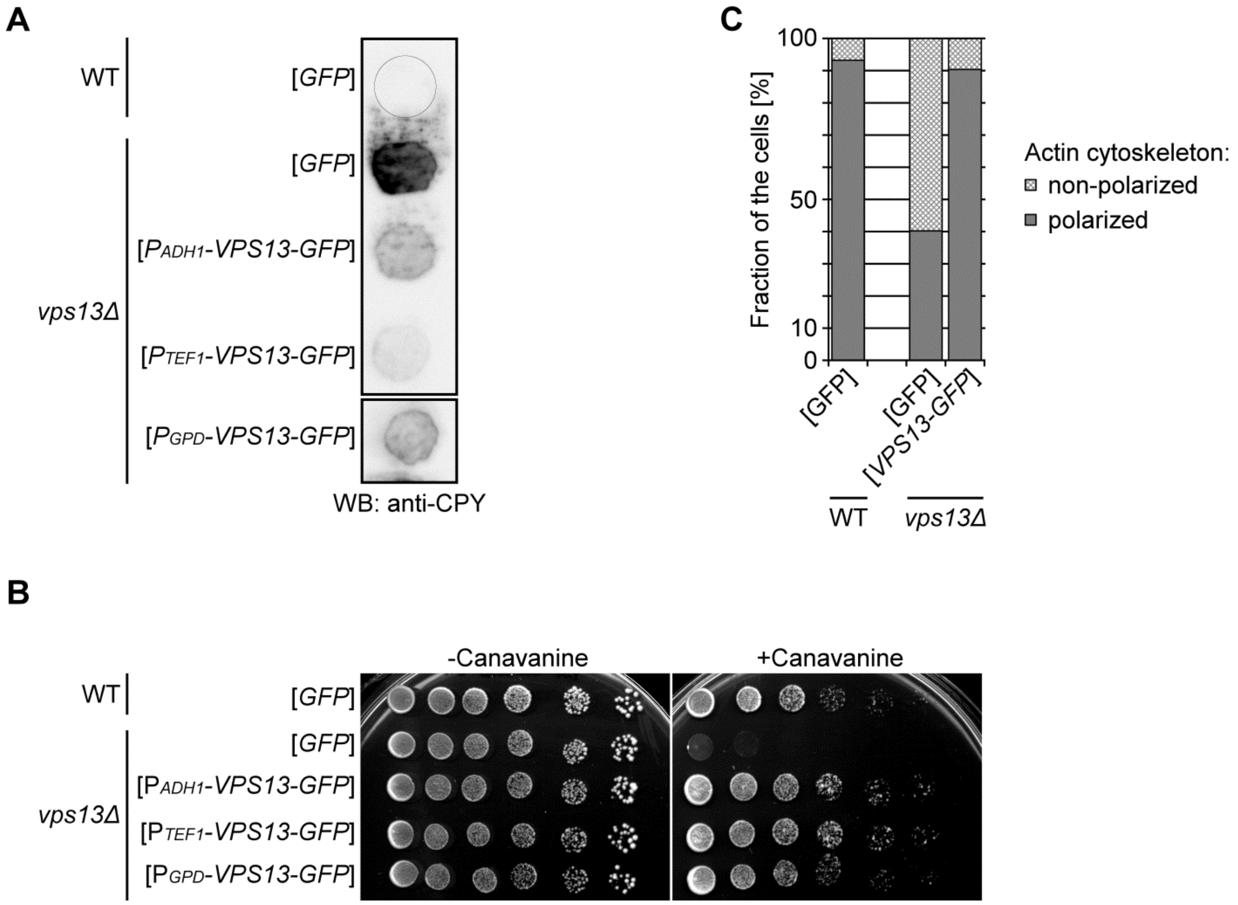

Figure S1. ***VPS13-GFP* under the transcriptional control of *ADH1*, *TEF1* or *GPD* promoter complements transport defects and improves actin cytoskeleton organization in *vps13Δ*. A**. Secretion of CPY. Yeast cultures were spotted on nitrocellulose membrane plated on SD-leu medium, incubated for 14-16 h and washed off. The level of secreted CPY was estimated using anti-CPY antibody. **B**. Sensitivity of yeast strains to canavanine. Serial dilutions of wild type and *vps13Δ* strains bearing indicated plasmids were spotted on synthetic minimal medium supplemented with (3 µg ml^{-1}) or without canavanine. **C**. Graphic representation of actin cytoskeleton organization in *vps13Δ* cells. The *vps13Δ* cells expressing P_{TEF1}-*VPS13-GFP* or bearing empty vector was grown to log-phase, fixed, stained using labeled phalloidine and observed by fluorescence microscopy. Wild-type cells bearing empty vector were used as a control. At least 100 cells were observed and the percentage of cells with well-polarized and non-polarized actin cytoskeleton is indicated.

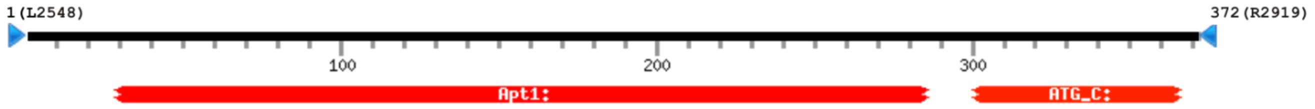


```
Query Vps13 (seq=LYKLKTASAT...SVTQDLEFQR Len=372 Neff=9.4  Nseqs=141)
Parameters  score SS:yes search:local realign with MAP:no

Hit                              Prob E-value P-value  Score    SS Cols Query HMM  Template HMM
PF10351 Apt1:  Golgi-body loca   99.7 5.9E-15 3.8E-19  141.2  25.1  222   27-283   164-477 (478)
PF09333 ATG_C:  Autophagy-rela   97.0 0.00077 4.9E-08   48.2   6.5   64  299-363    21-84  (96)
```

>[PF10351](PF10351) **Apt1 domain**: Golgi-body localisation protein domain. This domain is found in fungi, metazoans and plants. These include FMP27 and "maize" protein APT1 and *Arabidopsis* homologues SABRE and KIP. APT1 is required for pollen tube growth. It is a Golgi-localised protein and appears to regulate vesicular trafficking. SABRE and KIP are APT1 homologues and they are involved in the elongation of root cortex cells and pollen tubes respectively.

Probab=99.68 E-value=5.9e-15 Score=141.23 Aligned_cols=222 Identities=14% Similarity=0.176 Sum_probs=0.0

```
Q ss_pred                eccCccceeEEEEEEeCcEEEEEECCCCeEEEEEEEeeeEEEEEEc-CCCcEEEEEEEEEEEEEecccCCCCeEEEEeCC
Q Vps13          2574 QKDEDEKVNTQIVVSFKGVGISLINGRLQELLYINMRGIELRYNES-KAYQTFSWKMKWMQIDNQLFSGNYSNILYPTEI 2652(3144)
Q Consensus        27 ~~~~~~~~~~~~~l~l~~igiSlid~~~~El~~~~l~~i~~~~~~~~-~~~~~~~~l~V~~iQvDnq~~~a~~PVvL~~~~~  105 (372)
                     ....+....+.+.+.++.+...|+++....++-+.+.++.+.....  ++.....+.|+++|+-|..+++.||.+|.|.
T Consensus     2653 ~~~~~~~~~~~~~i~~~~i~w~ml~~~~~pf~~~~l~~~~f~r~~~~dgs~~n~l~I~~l~~~Nl~~~a~y~~il~p~--  241 (478)
T PF10351_consen  164 SKDKSPESALRWEICADEIIWHMLDDDGKPFLDLALSNFSFSRIESSDGSNSNRLEIGDLQVFNLLPNAVYPEILSPY--  241 (478)
T ss_pred                cccccCcccEEEEEEEcCeEEEEEEEcCCCCeeEEEEecCceeeeeecCCCcceEEEEEeEEEEEeCCCCCCCCccccCCC--


Q ss_pred                CCCccccccCCCEEEEEEEEeCCcccccccEEEEEEEEEeeEEEEEECHHHHHHHHHHhhCCCCccccCC-------------
Q Vps13           106 PYTEKEIENHPVISGSISKVNDSLQAVPYFKHVTLLIQEFSIQLDEDMLYAMMDFIKFPGSPWIMDS------------- 2719(3144)
Q Consensus       106 ~~~~~~~~~~~p~l~~~~~~~~~~~~~~~~~~~~~~~~~~v~l~pl~l~id~~~l~~l~~f~~~~~~~~~~~~~~~~------------  172 (372)
                      ........+|++++.+.+.++ .+++..++++.+.++|+.|++|+.+...|++|+.+-........
T Consensus      242 -~~~~~~~~~~~~~~~l~v~w~~~~p-VGGI~v~e~~ev~~~PL~iql~~~~~~~~l~~y~FP~~~~~~~~~~~s~~~~~~~~~~~~~~  319 (478)
T PF10351_consen  242 -SSSEMSSRKPMLRVYWRMLPP-VGGISVVEHFEVNLQPLKIQLEEETGKKLMEYFFPKRDEEKEESSMVKESSSDDDDD  319 (478)
T ss_pred                -CCCCccCCCcEEEEEEeecCC-CCCeeeeeeEEEEEeeeEEEehHHHHHHHHHHcCCCCcccccccccccCCccccccc


Q ss_pred                --------------------------------------------------------------------------------cc
Q Vps13          2720 --------------------------------------------------------------------------------RD 2721(3144)
Q Consensus       173 -------------------------------------------------------------------------------~~  174 (372)
                                                                                                     ..
T Consensus      320 ~~~~~~~~~~~~~~~~~~~~~~~~~~~~~~~~~~~~~~~~~~~~~~~~~~s~~~~~~~~~~s~~~~~s~~~~~~~~~~~~~~~~~~~~~  399 (478)
T PF10351_consen  320 SDDSSSSSSSSSSSSSSSSSSSSSSSSSRSSSLSSRLKPSSSSSSSPSDSSRSSSSSKSSWDESSIESKASSKPTSKRDS  399 (478)
T ss_pred                ccccccccccccccCCCCcccccccCCCCcchhhccCCCCCcccccCCCccccccCCccccccccccccccccccccccccc

                                             ┌I2749
Q ss_pred                cccccccCCCChhhhccCCCceeEEEEEEeCEEEEEEEEeCCCCCcccchhcccccccchhHHHHHHHHHhhceecCCCcEEEe
Q Vps13          2722 YKYDEEIQLPDVSELKTAGDIYFEIFHIQPTVLHLSFIRSDEISPGLAEETEESFSSSLYYVHMFAMTLGNINEAPVKVN 2801(3144)
Q Consensus       175 ~~~~~~~~~~l~~~~~~~~~~~~~~~~yi~~l~I~pi~l~lS~~~~~~~~~~~~~~~~~~~~~~~~~~~~l~~~~~~~l~~i~~a~l~l~  254 (372)
                     ...+        ....+..+.+.|.++.|+++.+.|||+..+..                          .+.++.|..|.+|
T Consensus      400 dd~~--------~M~~Ra~k~~~f~~ikI~~v~l~vSYkg~~~k-------------------------~l~dv~df~l~~P 2652(3144)
T PF10351_consen  400 DDID--------EMKERASKNFSFVYIKINSVVLCVSYKGPKSK-------------------------NLIDVHDFVLKLP  448 (478)
T ss_pred                ccHH--------HHHHhHHhccEEEEEEECcEEEEEEEecCCCCc-------------------------ccccccCcEEecC


Q ss_pred                eeeEeccccccHHHHHHHHHHHHHHHHHHH
Q Vps13          2802 SLFMDNVRVPLPILMDHIERHYTTQFVYQ 2830(3144)
Q Consensus       255 ~~~~~~~~~~~~t~~~L~~~~l~~~~Y~~q~~~q  283 (372)
                     .++++|..+|+.+|...++++.++.+++|
T Consensus      449 ~l~y~NktwS~~DL~~~lKkdiik~vL~h  477 (478)
T PF10351_consen  449 TLEYHNKTWSWLDLFMRLKKDIIKSVLSH  477 (478)
T ss_pred                CeEEEcCeecHHHHHHHHHHHHHHHHHHcC
```

Figure S2. **Prediction of the presence of APT1 domain in yeast Vps13 protein**. Analysis was performed using HHpred package (Söding et al., 2005; https://toolkit.tuebingen.mpg.de/hhpred) with standard parameters and PfamA (ftp://ftp.ebi.ac.uk/pub/databases/Pfam/) ver. 04-Jul-16 as a template HMM database. ***Upper panel***, graphical representation of predicted domain structure**.** ***Lower panel***, an alignment with secondary structure prediction of Vps13 region L2548-R2919 to a consensus sequence for the APT1 domain (PF10351). Additionaly, the position of isoleucine 2749 in Vps13 is indicated. Abbreviations: Q, query sequence; T, template sequence; ss_pred, secondary structure as predicted by PSIPRED (upper case letters: high probability, lower case letters: low probability; C, coil; E, β-sheet; H, helix). Symbols used in consensus line: "=", very bad match (column score below -1.5); "-", bad match (score between -1.5 and -0.5); ".", neutral match (score between -0.5 and +0.5); "+",good match (score between +0.5 and +1.5); "|", very good match (score above +1.5).

Söding J, Biegert A, Lupas AN (2005) The HHpred interactive server for protein homology detection and structure prediction. *Nucleic Acids Res* 33 (suppl 2), W244-W248.

Table S1. Mutant *vps13-I2749R* is defective in sporulation

Genotype	Independent diploid	Transformant	Cells	Tetrads	% of tetrads
vps13Δ/vps13Δ [—]	I	1	259	0	0
		2	306	0	0
		3	156	0	0
	II	1	321	0	0
		2	546	0	0
		3	384	0	0
vps13Δ/vps13Δ [*VPS13*]	I	1	320	144	45
		2	256	70	27
		3	166	43	26
	II	1	347	147	42
		2	359	151	42
		3	335	129	39
vps13Δ/vps13Δ [*vps13-I2749R*]	I	1	281	0	0
		2	198	0	0
		3	215	0	0
	II	1	373	0	0
		2	576	0	0
		3	457	0	0

Diploid strain from the cross of JK197-7B (*MAT***a** *vps13Δ::URA3 ura3-52 his3Δ200 met15Δ0 leu2,3-112*) with JK197-8C (*MAT*α *vps13Δ::URA3 ura3-52 lys2-801 trp1-1 leu2,3-112*) was obtained twice. Each diploid was transformed with empty vector [—] or plasmids bearing *VPS13* or *vps13-I2749R* allele. Three independent transformants were sporulated for one week at 28ºC. Full tetrads were counted twice for each culture.

**Table S2. Mutation *vps13-I2749R* is synthetically lethal with mutation *mmm1Δ***		
**Strain**	**Transformant**	**Full tetrads**
vps13Δ/VPS13 mmm1Δ/MMM1 [–]	1	3 PD
	2	1 PD
	3	3 PD
vps13Δ/VPS13 mmm1Δ/MMM1 [*VPS13*]	1	4 PD + 3 TT
	2	3 PD + 2 TT
	3	3 PD
vps13Δ/VPS13 mmm1Δ/MMM1 [*vps13-I2749R*]	1	1 PD
	2	6 PD
	3	5 PD

Diploid strain was obtained by crossing WR11 (*MATα LAS17-7Ala-mRFP::HIS3 ura3-52 leu2,3-112 lys2-801 his3Δ200 trp1-1 vps13Δ::URA3*) with BY4741 *mmm1Δ* (*MAT***a** *ura3Δ0 leu2Δ0 his3Δ1 met15Δ0 mmm1Δ::KanMX*). The diploid was transformed with empty vector [–] or plasmid bearing wild-type *VPS13* or mutated *vps13-I2749R* allele. Three independent transformants of each type were sporulated. Full tetrads were dissected and growth of spore clones was analyzed on SC-ura and YPD+G418 media to follow *vps13Δ::URA3* and *mmm1Δ::KanMX* presence. PD, parental ditype; TT, tetratype.
